# First-Step Results of Children Presenting with Bleeding Symptoms or Abnormal Coagulation Tests in an Outpatient Clinic

**DOI:** 10.4274/tjh.2013.0370

**Published:** 2015-12-03

**Authors:** İsmail Yıldız, Ayşegül Ünüvar, İbrahim Kamer, Serap Karaman, Ezgi Uysalol, Ayşe Kılıç, Fatma Oğuz, Emin Ünüvar

**Affiliations:** 1 İstanbul University İstanbul Faculty of Medicine, Department of Pediatrics, Division of Ambulatory Pediatrics, İstanbul, Turkey; 2 İstanbul University İstanbul Faculty of Medicine, Department of Pediatrics, Division of Pediatric Hematology and Oncology, İstanbul, Turkey; 3 İstanbul University İstanbul Faculty of Medicine, Department of Pediatrics, İstanbul, Turkey; 4 İstanbul University Institute of Child Health, Division of Ambulatory Pediatrics, İstanbul, Turkey

**Keywords:** children, Blood Coagulation, Hemophilia, Inherited coagulopathies, epistaxis, Menorrhagia

## Abstract

**Objective:**

Mild bleeding symptoms are commonly seen in the general population. The aim of this study was to determine the final clinical and laboratory features of children referred for a first evaluation with a suspected bleeding disorder in the pediatric outpatient clinic of İstanbul University.

**Materials and Methods:**

The medical records of 26,737 outpatients who were admitted to the Division of Ambulatory Pediatrics between 31 October 2011 and 31 October 2012 were evaluated retrospectively. Ninety-nine patients were initially diagnosed as having probable bleeding disorders and were followed up. The symptoms of bleeding in addition to coagulation tests were analyzed.

**Results:**

Of the 99 patients, 52 (52.5%) were male and 47 were female, and the mean age of the entire study group was 9.1±4.1 years (minimum-maximum: 2-18 years). Major bleeding symptoms were epistaxis in 36 patients (36.4%), easy bruising in 32 (32.3%), and menorrhagia in 6 (6.1%). After initial tests ordered by the pediatrician, 36 of 99 patients (36.4%) were diagnosed as having bleeding disorders that included von Willebrand disease in 12 (12.1%), hemophilia A or B in 9 (9.1%), and other rare factor deficiencies in 9 (9.1%). Six patients (6.1%) were found to have combined deficiencies. Seven of 36 patients had a family history of bleeding.

**Conclusion:**

Among the patients referred for bleeding disorders, 36.4% were diagnosed with a bleeding disorder with the help of primary screening tests ordered in the outpatient clinic.

## Abstract (tr)

**Amaç:**

Hafif kanama bozukluğu belirtileri toplumda sık görülmektedir. Bu çalışmanın amacı İstanbul Üniversitesi İstanbul Tıp Fakültesi Genel Pediatri Polikliniği’ne kanama bozukluğu şüphesi ile sevk edilen hastaların klinik ve laboratuvar özelliklerini belirlemektir.

**Gereç ve Yöntemler:**

31 Ekim 2011 ile 31 Ekim 2012 tarihleri arasında kanama bozukluğu şüphesiyle yönlendirilen 99 hastanın tıbbi kayıtları incelenmiştir. Başvuru semptomları ile pıhtılaşma testlerinin sonuçları değerlendirilmiştir.

**Bulgular:**

Olguların 47’si kız çocuğu olup ve yaş ortalaması 9,1±4,1 yıl (2-18 yıl) idi. Kanama semptomları 36 hastada (%36,4) burun kanaması, 32 (%32,3) hastada kolay morarma ve 6 hastada (%6,1) menoraji idi. Birinci basamak testleri sonrasında, 99 hastanın 36’sında (%36,4) primer kanama bozukluğu saptandı. Bunlardan 12’sinde (%12,1) von Willebrand hastalığı, 9’unda (%9,1) hemofili A veya B, 9’unda (%9,1) diğer nadir faktör eksiklikleri ve 6 hastada (%6,1) kombine faktör eksiklikleri saptandı. Otuz altı hastanın 7’sinde ailede kanama öyküsü vardı.

**Sonuç:**

Kanama bozukluğu şüphesi ile sevk edilen hastaların %36,4’ünde birinci basamak koagulasyon testleri ışığında kanama bozukluklarından biri saptandı.

## INTRODUCTION

When there is damage to the vascular wall, cessation of bleeding without interrupting the blood flow and maintenance of vascular integrity are ensured by hemostatic mechanisms. Hemostasis is a multifunctional physiologic mechanism involving the vascular wall, subendothelial tissues, platelets, coagulation factors in plasma, and fibrinolytic factors, where coagulants, anticoagulants, and fibrinolytic activities operate in balance [1,2,3].

Hemostatic disorders manifesting with bleeding may be caused by several factors including vascular issues, low platelet counts, platelet function disorders, and disorders of coagulation or fibrinolysis, which is due to either too much or too fast dissolving of blood clots [1,3].

A careful history and physical examination of a patient with bleeding symptoms leads to a correct diagnosis in 80%-90% of patients. Adequate laboratory tests are performed subsequently to confirm diagnosis [4,5,6].

In cases of bleeding disorders, the primary screening tests include complete blood count, peripheral blood smear, bleeding time test (if possible) using a platelet function analyzer (PFA-100), prothrombin time (PT), activated partial thromboplastin time (aPTT), thrombin time (TT), and fibrinogen levels [5,6]. Advanced tests are carried out later based on the pathological results from the primary screening tests. Regardless of whether primary screening test results are found to be normal, there may still be an underlying bleeding disorder. In these cases, factor 13 deficiency, von Willebrand disease (vWD) type 1, mild-type hemophilia A or B, mild factor 11 deficiency and mild deficiencies of other factors, alpha-2 anti-plasmin deficiency, plasminogen activator inhibitor-1 deficiency, collagen tissue diseases, vitamin C deficiency, and various vascular bleeding disorders should be considered [4,6].

Mild bleeding symptoms such as epistaxis, easy bruising, gingival bleeding, and prolonged menstrual bleeding are commonly seen in the general population and reported in up to 25%-45% of healthy people [7]. Although patients who present with these symptoms may have underlying bleeding disorders, initial tests for bleeding etiology may yield normal results [8,9].

The purpose of this study was to evaluate patients who were referred to the Division of Ambulatory Pediatrics with suspected bleeding disorders.

## MATERIALS AND METHODS

A total of 26,737 outpatients were admitted to the İstanbul Faculty of Medicine’s Department of Pediatrics from 31 October 2011 to 31 October 2012. After exclusion of all patients with immune thrombocytopenia, 115 patients with suspected bleeding disorders were evaluated retrospectively. Thirteen of these patients were not included because of known bleeding disorders or they were lost during follow-up. Three patients were excluded from the study after they were diagnosed as having secondary thrombocytopenia caused by viral infections or platelet function disorder. Thrombocytopenia and platelet function disorders were not included in the evaluation.

**Figure 1:**
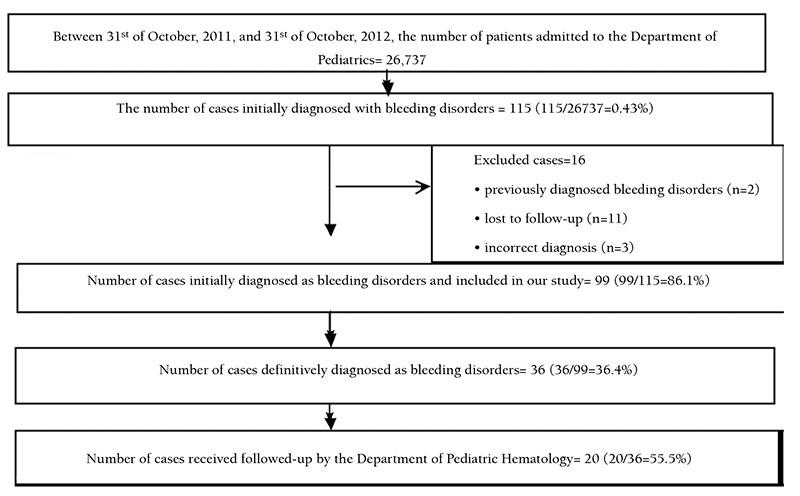
Study flow-chart.

This study was thus conducted with 99 patients (Figure 1).

All the admission symptoms, history, physical examination findings, laboratory test results, and initial and definitive diagnoses are based on the database from the hospital’s automation system and the patients’ charts.

We recorded the patients’ sex, age, symptoms, site of bleeding, duration of hemorrhage, existence of any bleeding problems in the newborn period, and previous personal or family history of bleeding disorders.

The first primary tests performed on patients suspected of having a bleeding disorder were complete blood count, bleeding time or PFA-100, PT, aPTT, TT, and fibrinogen levels. A peripheral blood smear was performed in all patients to evaluate platelet count and size, thus excluding pseudothrombocytopenia. Bleeding time was measured either in vitro with the PFA-100 (n=61) or in vivo by Duke’s method (n=5). Advanced laboratory investigations were performed on all patients whose initial tests revealed any pathological findings. VWF antigen, ristocetin cofactor activity (Ricof), and factor level (II, V, VII, VIII, IX, X, XI, XII, XIII) tests were performed in the Pediatric Hematology Hemostasis Laboratory.

The patients with abnormal test results were referred to the Pediatric Hematology and Oncology Unit for advanced evaluation and follow-up. Tests with abnormal results were repeated again at the next visit. The patients’ folders that were created in the Pediatric Hematology and Oncology Unit were evaluated for the definite diagnosis. Initial and definitive diagnoses of these patients were recorded.

## RESULTS

A total of 26,737 outpatients were admitted to our unit during the 1-year period of study. Ninety-nine (0.37%) patients were initially diagnosed with probable bleeding disorders and were followed up. Fifty-two (52.5%) patients were male and 47 (47.5%) were female, and their mean age was 9.1±4.1 years (minimum-maximum: 2-18 years).

The most frequent symptoms were epistaxis in 36 of the patients (36.4%), easy bruising in 32 (32.3%), prolonged and/or massive menstrual bleeding in 6 (6.1%), and gingival bleeding in 2 (2%) (Table 1). Duration of the symptoms ranged from 2 days to 6 years.

According to the laboratory test results, 63 of the patients (63.6%) had no bleeding disorders, whereas 36 (36.4%) were diagnosed with bleeding disorders (Figure 1). The final diagnosis included vWD type 1 in 8 (8.1%); vWD type 2 in 4 (4%); mild hemophilia A in 4 (4%); vWD type 1 and FXI deficiency in 3 (3%); FV deficiency in 3 (3%); moderate hemophilia A in 2 (2%); hemophilia A carrier in 2 (2%); FVII deficiency in 2 (2%); FXI deficiency in 2 (2%); FX deficiency in 1 (1%); FXII deficiency in 1 (1%); combined FII, VII, IX, X, and FXII deficiency in 1 (1%); combined FV and FVIII deficiency in 1 (1%); combined FVII and FX deficiency in 1 (1%); and hemophilia B carrier in 1 (1%) (Table 1).

Seven (19.4%) of 36 patients who were diagnosed with bleeding disorders had a family bleeding history. Family histories of the patients for coagulation disorders are presented in Table 2.

Twenty-eight percent of the patients with epistaxis (10 of 36 patients; 3 cases of mild hemophilia A, 2 of vWD type 1, 1 of vWD type 2, 1 of vWD type 1+FXI deficiency, 1 of FVII deficiency, 1 of FV deficiency, and 1 of combined FII, VII, IX, X, and XII deficiency), 28.1% of the patients with easy bruising (9 of 32 patients; 3 cases of vWD type 1, 1 of vWD type 2, 1 of a hemophilia A carrier, 1 of FV deficiency, 1 of FXI deficiency, 1 of FVII+X deficiency, and 1 of FXII deficiency), and 33.3% of the patients with menorrhagia (2 of 6 patients; 1 a hemophilia B carrier and 1 with FX deficiency) were diagnosed with a bleeding disorder after the first evaluation due to clinic and laboratory results.

Unfortunately, only 20 of 36 patients with bleeding disorders could be evaluated in the Division of Pediatric Hematology and Oncology. When these 20 patients were evaluated again, 16 of them (16/20, 80%) were confirmed to have the same diagnosis that the general pediatrician had established, whereas 4 of them had different diagnoses. One patient with factor V and VIII deficiencies at the Division of Ambulatory Pediatrics was diagnosed with factor V deficiency at the Division of Pediatric Hematology and Oncology. Another patient with probable vWD type 1 and factor XI deficiency was diagnosed with vWD type 1. Furthermore, a patient with probable mild-type hemophilia A and a probable hemophilia A carrier were not diagnosed with a bleeding disorder after the evaluation in the Division of Pediatric Hematology and Oncology (Table 3).

## DISCUSSION

The first step for a patient with a suspected bleeding disorder is to get a detailed medical history, such as initial time of bleeding; history of any traumas; patient’s operation history; circumcision history for male patients; any known liver, kidney, or malabsorption-related disorders; and the history of bleeding disorders in other family members. In physical examination, location and type of the bleeding and any accompanying signs should be investigated. First diagnosis can be made for most of the patients with careful medical history and physical examination, and final diagnosis can be made with laboratory tests [1,2,3].

The most common congenital coagulation disorders in childhood are vWD, hemophilia A and B, and factor XI deficiency [1,10]. Nevertheless, rare factor deficiencies may also be seen in childhood [11]. In our study, the patients who had bleeding disorders were mostly diagnosed with vWD, mild-type hemophilia A, and factor XI deficiency. In this study, vWD was the most frequently diagnosed disease, in accordance with the literature. The relatively high rate of rare factor deficiencies may be explained by consanguinity of parents. In addition, our university’s hospital is a tertiary hospital.

The most commonly seen symptoms in patients with bleeding disorders are easy bruising, recurrent epistaxis, prolonged bleeding after circumcision or teeth extraction, menorrhagia, and hemarthrosis [1,4,8,12]. However, these symptoms may vary by age. For example, in the neonatal period, umbilical bleeding, cephalic hematoma, and hematoma and ecchymosis at injection sites can be seen; in infants, mucosal bleeding, easy bruising, and hemarthrosis when the child starts to walk; and in older children, bleeding after surgical procedures [1,7,10]. Nose, skin, and oral mucosal bleedings are easily recognized by parents, whereas gastrointestinal and genitourinary bleeding may not be easily noticed. Therefore, anamnesis is very important. In our study, the most commonly seen symptoms were epistaxis, easy bruising, menorrhagia, and gingival bleeding. Epistaxis is a symptom commonly seen in the general population. Epistaxis may be a symptom of a bleeding disorder, but it may also be due to trauma, nose-picking, sinusitis, rhinitis, nasal polyps, and high blood pressure. In patients manifesting with recurrent epistaxis, the rate of detecting a bleeding disorder is 5.5%-33% [13,14]. In our study, of all of patients who were diagnosed with a bleeding disorder, 28% of them had epistaxis. These findings are consistent with the literature data [14].

The rate of bleeding disorders in adult patients with menorrhagia is 15%, but in adolescent patients the rate goes up to 10%-40% [15]. In our study, 2 patients out of 6 (33.3%) with menorrhagia had a bleeding disorder. Therefore, in adolescents with menorrhagia, existence of an underlying bleeding disorder should be investigated. The final diagnosis was different in 4 cases. This shows that a second laboratory evaluation should be done for all patients with bleeding symptoms [16].

In conclusion, rational approaches to children who present with bleeding symptoms require detailed history taking and careful physical examination, followed by adequate laboratory tests to confirm the initial diagnosis. In this study, about 40% of the children presenting with bleeding symptoms were diagnosed with a bleeding disorder in our outpatient clinic after the first evaluation. Additionally, an underlying bleeding disorder should be considered in a child with menorrhagia.

## Figures and Tables

**Table 1:**
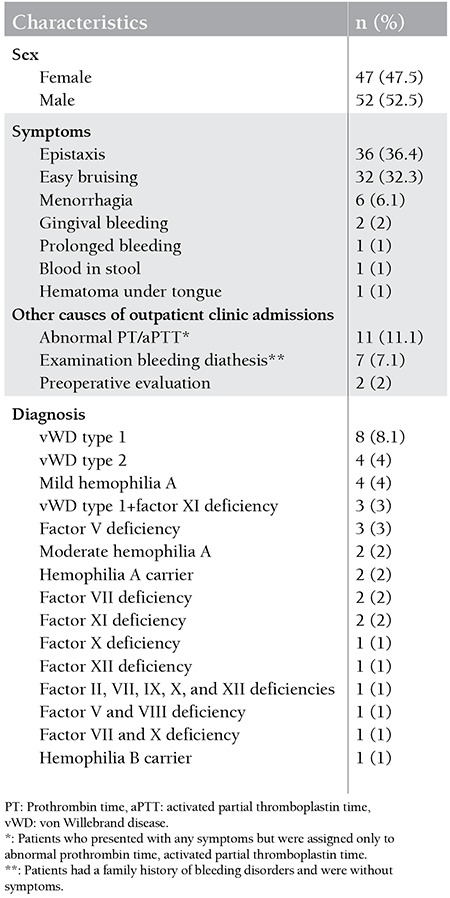
Characteristics of patients initially diagnosed with bleeding disorders (n=99).

**Table 2:**
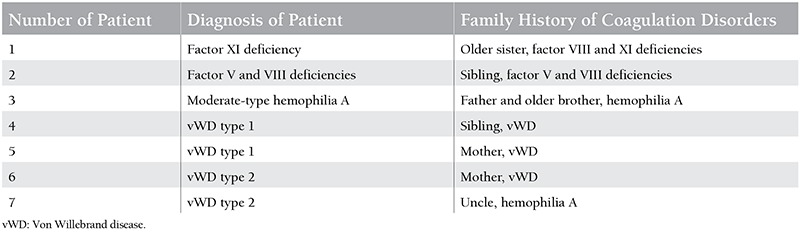
Family history of the patients for coagulation disorders.

**Table 3:**
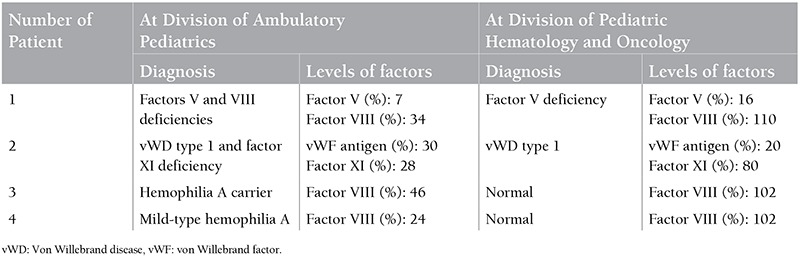
Laboratory test results of the patients diagnosed with different bleeding disorders.
